# Concurrent Chronic Myeloid Leukemia and Metastatic Renal Cell Carcinoma in a 61-year-old Female: Successful Treatment with Low-Dose Immunotherapy and Combination Targeted Therapy

**DOI:** 10.15586/jkc.v13i2.437

**Published:** 2026-05-29

**Authors:** Ajay Gupta, Shuaib Zaidi, Uma Ravishankar, Shefali Kalra, Sangeeta Taneja, Pankaj Baweja

**Affiliations:** 1 Department of Medical Oncology, Indraprastha Apollo Hospital, Jasola, New Delhi, India;; 2 Department of Surgical Oncology, Indraprastha Apollo Hospital, Jasola, New Delhi, India;; 3 Department of Nuclear Medicine, Indraprastha Apollo Hospital, Jasola, New Delhi, India;; 4 Department of Radiology, Indraprastha Apollo Hospital, Jasola, New Delhi, India;; 5 Department of Pathology, Indraprastha Apollo Hospital, Jasola, New Delhi, India

**Keywords:** axitinib, chronic myeloid leukemia, dasatinib, nivolumab, renal cell carcinoma

## Abstract

We report a rare case of a 61-year-old female diagnosed with concurrent Chronic Myeloid Leukemia (CML) and Renal Cell Carcinoma (RCC). The patient had a history of CML treated with imatinib for 4 years, with loss of complete hematological response for 3 months before being diagnosed with RCC and lung metastases. Due to a T315I mutation in the BCR-ABL1 gene, the treatment regimen included a novel combination of Axitinib, Dasatinib, and low-dose nivolumab. The patient showed a remarkable therapeutic response with a complete metabolic response accompanied by a highly significant reduction in the size of the tumor and complete resolution of the metastatic lung lesions, as well as a major molecular response in terms of CML disease control.

## Introduction

Concurrent hematologic and solid malignancies are uncommon, and their management poses significant challenges. Chronic myeloid leukemia (CML) is a myeloproliferative neoplasm characterized by the presence of the Philadelphia chromosome (BCR-ABL1 fusion gene). Renal cell carcinoma (RCC) is the most common type of kidney cancer and can present de novo as metastatic disease. This report explores the therapeutic approach and outcomes in a patient with concurrent CML and metastatic RCC.

## Report

A 61-year-old female was diagnosed with chronic CML 4 years ago at an outside hospital. Peripheral blood examination revealed moderate anemia with a hemoglobin level of 9.0 g/dL. The total leukocyte count was markedly elevated at 65,500/µL, consistent with significant leukocytosis.

Differential leukocyte count demonstrated a predominance of mature granulocytes, with segmented neutrophils comprising 89% and band forms 9%, indicating a marked left shift. Immature myeloid precursors were present, including metamyelocytes (2%), myelocytes (1%), and promyelocytes (2%). Eosinophils were increased (4%), and no significant increase in blast percentage was noted in the peripheral smear. Platelet count was elevated at 502,000/cu mm, suggestive of thrombocytosis. She also had a splenomegaly of 5 cm below the left costal margin and an intermediate Sokal Risk Score.

The bone marrow biopsy revealed a hypercellular marrow with complete obliteration of the fat spaces, a myeloid to erythroid ratio of 11:1, marked hyperplasia of granulocytic cells with maturation, 12% (CD34 positive) blasts, and an increased number of megakaryocytes with the presence of small hypolobated forms. The diagnosis was confirmed by Reverse Transcription Polymerase Chain Reaction (RT PCR) for BCR-ABL1 fusion gene transcript (p210) on the peripheral blood and by cytogenetic analysis on the bone marrow that showed t(9;22) translocation. She was started on treatment with Imatinib at a dose of 400 mg/day and was in complete molecular response till a year back, when her last test was done. She then ceased following up with her clinic but took the medications regularly. She presented with complaints of hematuria and right flank pain to the hospital in late 2023, and was diagnosed with a renal mass on ultrasound and was investigated further.

**Figure 1:**
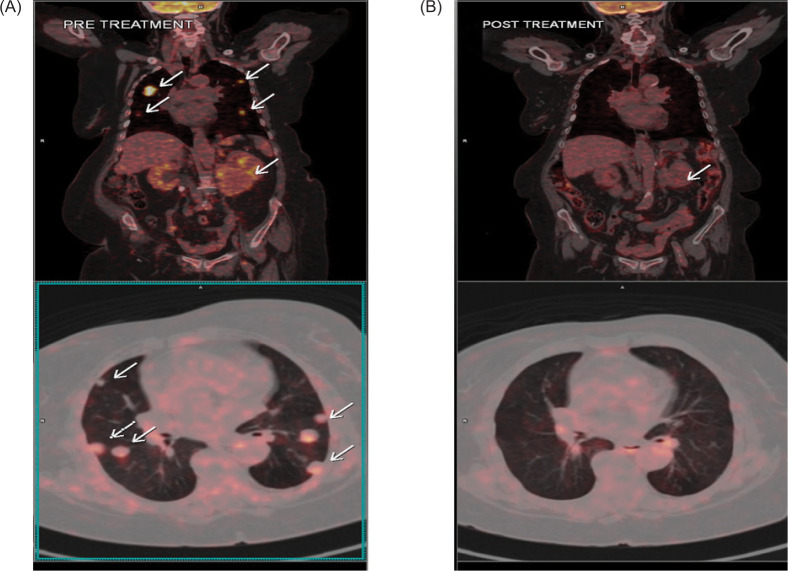
(A) Pretreatment: FDG PET-CT scan showing FDG-avid mass lesion (SUV max 15.31) arising from the lower pole of the right kidney measuring 8.0 x 6.8 x 7.7 cm, accompanied by multiple FDG-avid (SUV max 31.40) and non-avid lung metastases scattered in both lungs, with the largest lung lesion seen in the right upper lobe measuring 2.3 x 1.4 cm. (B) Posttreatment: FDG PET-CT scan done 16 months after initiation of treatment showing complete metabolic resolution and reduction in size of the renal mass (3.2 x 2.1 cm in size, Non-FDG-avid). Regression of the renal mass was observed, with near complete resolution of the lung metastases (non-FDG avid).

An 18-Fluoro Deoxy-Glucose Positron Emission Tomography Computed Tomography (18 FDG PET-CT) revealed an 8.0 x 6.8 x 7.7 cm, FDG-avid mass lesion (Standardized Uptake Value maximum, SUV max: 15.31) arising from the lower pole of the right kidney, as well as multiple lung metastases (SUV max 31.40), the largest lung lesion being 2.3 x 1.4 cm. The laboratory investigations indicated a hemoglobin level of 9.1 g/dL, a total leukocyte count (TLC) of 26,000/cu mm, and a platelet count of 607,000/cu mm (Figure 1A). Further molecular analysis revealed elevated BCR-ABL1 transcripts at 56.05% on the International Scale, along with a T315I mutation.

**Figure 2:**
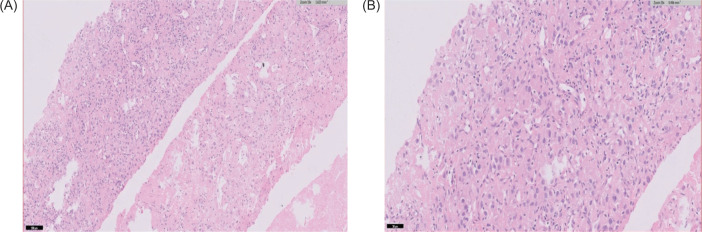
Nests and sheets of cells noted with distinct membrane and granular eosinophilic cytoplasm, suggestive of clear cell renal carcinoma in 10x (A) and 40x magnifications (B) (H&E).

The biopsy of the renal mass was suggestive of clear cell RCC (Figure 2). The lung masses were not biopsied and were presumed to be metastatic based on the findings of the PET scan. The patient was started on parenteral nivolumab 40 mg every 3 weeks because of financial constraints, and oral axitinib 5 mg/day and dasatinib 70 mg/day because of the unavailability of ponatinib and asciminib.

Within 2 weeks of treatment initiation, the patient developed severe hypertension, necessitating a reduction in the dose of axitinib to 2.5 mg/day. After the dose reduction, the patient tolerated the therapy well without any side effects. She was advised to follow up with routine blood tests: hemogram, kidney, liver function tests, thyroid function tests, fasting blood glucose and serum cortisol every 3 weeks; X-ray of the chest every 2 months; and peripheral blood RT-PCR analysis of BCR-ABL p210 transcript every 3 months. A follow-up PET-CT at 3 months demonstrated a reduction in the size of the renal mass and the metastatic lung lesions. Hematological parameters showed significant improvement with an Hb level of 10.1 g/dL, TLC of 7400/cu mm, and platelet count of 430,000/cu mm.

At the 18-month follow-up, the PET-CT scan revealed further regression in the size of the renal mass (3.3 x 2.1 cm) with complete metabolic response and near total resolution of the lung metastases (Figure 1B). The hemogram revealed an Hb level of 10.6 g/dL, TLC 6200/cu mm, and platelet count 301,000/cu mm with normal differentials, and the BCR-ABL1 transcript level was less than 0.006%, suggestive of a major molecular response.

## Discussion

Axitinib is an ATP-competitive inhibitor of vascular endothelial growth factor receptors (VEGFR) 1, 2, and 3 (1, 2). It acts on other receptors, such as KIT and platelet-derived growth factor receptor (PDGFR) (2). It is a second-generation tyrosine kinase inhibitor (TKI) and has significant activity in RCC, especially in combination with checkpoint inhibitors, namely pembrolizumab and nivolumab (3, 4).

Axitinib, primarily a VEGFR inhibitor, has demonstrated off-target inhibition of the BCR-ABL1 T315I mutation. Axitinib inhibited the BCR-ABL (T315I) mutation in samples from patients with CML and Philadelphia chromosome–positive B-cell acute lymphocytic leukemia (Ph+ ALL). Tyrosine kinase activity of the T315I mutation was inhibited at a very low concentration of axitinib (100 nM), which compared favorably with the concentration required to inhibit the tyrosine kinase activity of VEGFR (20 nM). The activity of wild-type BCR-ABL kinase was inhibited with a significantly higher concentration of axitinib (3800 nM), suggesting that axitinib specifically and potently inhibits BCR-ABL (T315I). Compared with other BCR-ABL kinase inhibitors (such as imatinib and dasatinib), axitinib was shown to fill different binding spaces, making the bond to the T3151 protein tighter (5).

This case report described how, after 2.5 years of TKI treatment, including imatinib and dasatinib, resistance due to the development of T315I mutation was tackled by the combined application of dasatinib and axitinib, resulting in an excellent and early molecular response within 2 months (6).

Dasatinib is a potent multikinase inhibitor targeting BCR–ABL, the SRC family of kinases (SRC, LCK, HCK, YES, FYN, FGR, BLK, LYN, FRK), receptor tyrosine kinases (c-KIT, PDGFR, DDR1 and 2, c-FMS, ephrin receptors), and TEC family kinases (TEC and BTK) and demonstrates activity against most imatinib-resistant BCR–ABL mutations. It is, however, ineffective against T315L mutations (7).

Dasatinib notably suppresses RCC cell survival by inducing G1/S cell cycle arrest. Experimental studies have shown that it may serve as a powerful drug candidate to treat subgroups of RCC patients with hyperactivated Src-YAP signaling axis, and the alteration of p-YAP could serve as a functional response biomarker of dasatinib in RCC (8).

Cabozantinib and dasatinib synergize to induce tumor regression in non-clear cell RCC in experimental models (9). However, their combined use can be associated with adverse drug interactions, which precluded their use in our case (10).

Drug interactions are a challenge when managing patients with two TKIs. Dasatinib could potentially increase the levels of axitinib by affecting the hepatic or intestinal enzyme CYP3A4 metabolism. In fact, we had to reduce the dose of axitinib to 2.5 mg/day on account of the severe hypertension, but it is hard to say whether it was the effect of axitinib alone or because of some drug interactions. Nivolumab is an effective drug in RCC with single-agent response rates of 25% and a median overall survival of 25 months (11). Combinations of nivolumab, cabozantib, and axitinib are well studied and very effective in both first- and second-line settings (4, 12)

Low-dose nivolumab was chosen in this case because of financial constraints, but it is effective in RCC. In a large Phase 1b, open-label, dose-escalation study of nivolumab at doses ranging from 0.1 to 10 mg/kg administered every 2 weeks in 306 patients with advanced malignancies, the safety profile and objective response rates (ORRs) were similar across tumor types and all doses (0.1–10 mg/kg) in RCC, respectively (13).

Concurrent occurrence of CML and RCC is uncommon. A review of the literature has revealed six such cases (14).

Choosing the correct therapeutic modality in this scenario of dual malignancies (RCC and CML) can be immensely challenging. Dasatinib, a drug for CML, also has action against RCC, and while in combination with axitinib (a potent drug against RCC), it can also be used to tackle T315l mutations in CML, which are resistant to dasatinib (6–9). Nivolumab in combination with TKIs such as axitinib is one of the regimens used in the treatment of metastatic RCC (4). Low-dose nivolumab can also be effective in RCC (11–13). This rationale was used to devise a treatment regimen for the patient. The synergistic effect of axitinib and dasatinib, in combination with immune modulation by nivolumab, resulted in excellent clinical outcomes in this case.

## Conclusion

This case underscores the importance of individualized treatment strategies in patients with concurrent hematological and solid malignancies. Multimodal therapy involving TKIs and immune checkpoint inhibitors can yield favorable responses, even in the presence of drug resistance mutations.

## Consent

Written informed consent was obtained from the patient for publication of the report and accompanying images.

## Mandatory Disclosure on Use of Artificial Intelligence

The authors declare that no AI-assisted tools were used in the preparation of this manuscript. All references have been manually verified for accuracy and relevance.
